# The mediating role of self-efficacy in the association between family support and mental health among Taiwanese adolescents and youths in low-income families: a half-longitudinal mediation model

**DOI:** 10.1186/s12889-026-28668-4

**Published:** 2026-07-24

**Authors:** Ching-Wen Chang, Ling Lin, Ji-Kang Chen

**Affiliations:** 1 National Taiwan Normal University, Taipei, Taiwan; 2 The Chinese University of Hong Kong, Shatin, Hong Kong

**Keywords:** Adolescent, Youth, Positive Psychology, Mental health, Psychological distress, Life satisfaction, Self-efficacy, Social support, Family support

## Abstract

**Background:**

Previous studies have examined the correlation between social support and adolescent mental health, as well as the mediating role of self-efficacy in this association, primarily using cross-sectional data. However, few have employed longitudinal designs, considered both positive and negative aspects of mental health, or focused specifically on adolescents from financially strained families. This study aimed to investigate the mediating role of self-efficacy in the relationship between family support and both positive and negative dimensions of mental health among Taiwanese adolescents and youths from low-income families.

**Methods:**

Using two-wave longitudinal panel data, the study included 1,792 economically disadvantaged adolescents and youths across 23 counties and cities in Taiwan. Life satisfaction and psychological distress were used to indicate positive and negative dimensions of mental health, respectively. A half-longitudinal mediation analysis was conducted.

**Results:**

Family support at T1 was positively associated with self-efficacy and life satisfaction but not psychological distress at T2. Self-efficacy at T1 was significantly related to life satisfaction but not psychological distress at T2. The half-longitudinal mediation analysis further supported the mediating role of self-efficacy in the association between family support and life satisfaction.

**Conclusions:**

Family support was predictive of positive, but not negative, aspects of mental health. In addition, self-efficacy served as a mediator in the link between family support and positive mental health. These findings underscore the importance of policies and interventions that strengthen both family support and self-efficacy while also highlighting the need for differentiated approaches to address the positive and negative dimensions of mental health among adolescents and youths from low-income families in Taiwan.

## Background

Adolescents and youths from low-income families are at greater risk of poor mental health due to multiple interconnected factors related to socioeconomic status [1]. Limited financial resources can restrict access to essentials such as nutritious food, safe and stable housing, and health care, which can negatively impact development and mental well-being [2, 3]. Financial strain can also lead to feelings of social exclusion, shame, and lack of control over life circumstances, which can heighten emotional distress [4]. Furthermore, low-income status may increase adolescents’ and youths’ exposure to violence, discrimination, and social mistrust, making them more vulnerable to mental health issues [5]. Numerous studies have demonstrated a significant association between poverty and poor mental health within this population [6, 7]. Findings from the *Survey on Adverse Childhood Experiences*conducted in Taiwan between 2024 and 2025 indicate that children and adolescents from economically disadvantaged backgrounds continue to experience substantial emotional distress, with 59.7% reporting depressive or low mood [8]. Given this heightened risk, it is crucial to identify factors that can help mitigate poor mental health outcomes and enhance overall mental well-being in adolescents and youths from low-income families in Taiwan.

Family support has been identified as a protective mental health factor in adolescents [9, 10]. For example, emotional warmth and understanding from family members help alleviate feelings of loneliness and stress [11]. Parental support also fosters supportive parent–child relationships and enhances adolescents’ emotional regulation, which in turn promotes improved psychological functioning and mental health outcomes [11–13]. Moreover, perceived social support from family reduces adolescents’ likelihood of experiencing anxiety and depressive symptoms [14].

The literature suggests that self-efficacy may be a key pathway through which family support exerts its impact on mental health [15, 16]. Self-efficacy refers to an individual’s belief in their capacity to manage challenges, conduct tasks, and achieve success [17]. Family members can strengthen self-efficacy by modeling effective coping strategies; offering encouragement, comfort, and guidance; and reinforcing the belief that challenges are manageable [18]. Support provided by family members can also help individuals to achieve personal goals, thereby enhancing their self-efficacy, which in turn facilitates adaptive adjustment and promotes overall psychological well-being [18, 19].

According to the World Health Organization, mental health is a “state of well-being in which an individual realizes their own abilities, can cope with the normal stresses of life, can work productively, and is able to contribute to their community” [20]. This definition presents mental health as a multifaceted concept that extends beyond the mere absence of mental illness and comprises both positive and negative aspects. On the positive side, indicators like life satisfaction are commonly used to assess individuals’ well-being [21]. In contrast, the negative dimension encompasses a broad spectrum of mental health issues, including the presence of mental illness and elevated levels of psychological distress [22]. Research has demonstrated that the positive and negative dimensions of mental health are distinct yet interrelated constructs [21, 22]. Accordingly, the effects of social support may differ across positive and negative dimensions of mental health.

However, research among adolescents from the general population has demonstrated that social support is associated with both positive mental health and negative mental health outcomes [10, 14, 18, 23]. Whether family support exerts comparable effects on positive and negative mental health remains unclear among Taiwanese adolescents and youths from low-income families. In Taiwan, where collectivism and Confucian values are central, family members are strongly bonded and are obligated to support each other [24]. Hence, family support is intertwined with strong role expectations and a sense of obligation [25, 26]. Rather than being solely a source of emotional or instrumental resources, family support is embedded in a relationship structure of obligation and reciprocity. Accordingly, when adolescents receive family support, they may simultaneously experience a stronger sense of obligation to meet family expectations or contribute to the family. This dynamic may be particularly salient among adolescents from low-income families, where the use of limited family resources can further intensify psychological pressure [27]. Furthermore, the high value placed on "escaping poverty through education" in Taiwan turns academic performance into a primary way of fulfilling family responsibilities [28]. Due to limited resources and environmental constraints, adolescents from low-income families may feel more frustration and pressure when facing this responsibility. Taking into account culturally embedded expectations, obligations, and resource constraints, family support may function more as a promotive factor that enhances positive aspect of mental health, while having a more limited effect on alleviating negative mental health outcomes.

In contexts shaped by both cultural expectations and economic disadvantage, the relationship among family support, self-efficacy, and adolescent mental health, as suggested by prior literature, would be more complex. In the Taiwanese cultural context, which emphasizes collective family interests and functional roles, family support often takes a goal-oriented form, with parents often overly involved in their children’s decisions [29, 30]. Although such involvement is typically well intentioned, it may undermine adolescents’ autonomy, making it more difficult to develop stable self-efficacy [31]. For those from low-income families, prolonged exposure to limited resources and restricted opportunities may also hinder their ability to pursue and achieve goals, thereby diminishing self-efficacy [32, 33]. At the same time, growing up in socioeconomically disadvantaged environments can also reduce adolescents’ perceived control over their lives, further contributing to lower self-efficacy [34–36]. Under these conditions, family support may not effectively translate into improved mental health outcomes, in part because its influence on self-efficacy is constrained. As a result, even when adolescents perceive high levels of family support, it is unclear whether this support can be effectively translated into self-efficacy, or whether self-efficacy serves as a mediating mechanism between family support and mental health.

### Sex differences

Prior research suggests that girls tend to be more sensitive to relational stress and place greater emphasis on interpersonal relationships [37]. Hence, family support may function more directly in influencing their mental health. In contrast, boys may place greater emphasis on competence [38], making self-efficacy a more central pathway through which family support influences mental health. Accordingly, it is reasonable to speculate that self-efficacy may play a weaker mediating role in the relationship between family support and mental health for girls.

### The current study

The present study aimed to examine the association between family support and mental health, investigate the mediating role of self-efficacy in this association among Taiwanese adolescents and youths from low-income families, and explore whether these relationships differ by sex. There are three research questions:

1. Is family support associated with later positive and negative mental health?
2. Does self-efficacy mediate the association between family support and later positive and negative mental health?
3. Are there sex differences in the mediating role of self-efficacy in the association between family support and later positive and negative mental health?

Although social support has been associated with better mental health among adolescents [10, 14, 18, 23], it remains unclear whether family support exerts similar influences on positive and negative dimensions of mental health among adolescents and youths from low-income families in Taiwan. This question is particularly important given the combined influence of chronic economic hardship and cultural values emphasizing family obligation, interdependence, and reciprocity, which may shape how family support is experienced and translated into mental health outcomes. In addition, the mediating role of self-efficacy and potential sex differences in these relationships remain underexplored. Using longitudinal data, the present study addresses these gaps by providing a more rigorous examination of the temporal relationships among family support, self-efficacy, and mental health than is possible with cross-sectional designs. The study contributes to a better understanding of the mechanisms through which family support may influence positive and negative mental health over time among socioeconomically disadvantaged adolescents and youths in Taiwan.

## Methods

This study employed secondary data analysis of the Taiwan Database of Children and Youth in Poverty (TDCYP) [39], the result of a panel study conducted by the Taiwan Fund for Children and Families (TFCF). The TDCYP was established to investigate the experiences and outcomes of children and adolescents in poverty, defined by the TFCF as those from families unable to meet basic living needs or maintain normal daily functioning. Utilizing a systematic sampling method, the TDCYP includes a representative sample of economically disadvantaged children and adolescents selected from 6,427 TFCF-supported individuals, aged 7 to 22, living in 23 counties and cities throughout Taiwan. The TDCYP comprises five waves of data collected between 2009 and 2017, with a 2-year interval between each round of data collection. Questionnaires for participants aged 12 and below were administered through face-to-face interviews conducted by trained interviewers, whereas questionnaires for adolescents aged 13 and above were self-administered. The current analysis is based on data from Wave 2 (2011, T1) and Wave 3 (2013, T2), the only waves including information on all study variables. In the TDCYP, 1,792 adolescents aged 13 and above completed surveys in both waves and were selected for the current analysis.

### Measures

Mental health was operationalized through a dual-factor lens. Positive mental health is commonly conceptualized as a state of well-being [20]. Because life satisfaction reflects the cognitive component of subjective well-being and is widely recognized as a key indicator of positive mental health [21], it was used as the indicator of positive mental health in this study. Meanwhile, psychological distress, served as the indicator for negative mental health. This approach allows for the examination of whether family support differentially predicts the enhancement of well-being versus the alleviation of distress.

Positive mental health (Life satisfaction). Life satisfaction, an indicator of positive mental health in the present study, was measured using the Satisfaction With Life Scale [40]. It comprises five items (e.g., “In most ways my life is close to my ideal,” “The conditions of my life are excellent”). The scale uses a 7-point Likert scale from 1 (*strongly disagree*) to 7 (*strongly agree*), with a higher score indicating a higher level of life satisfaction. Cronbach’s alphas of this scale were 0.83 at T1 (0.83 for boys and 0.84 for girls) and 0.83 at T2 (0.83 for boys and 0.83 for girls).

Negative mental health (Psychological distress). Psychological distress, an indicator of negative mental health, was evaluated using five items of the Brief Symptoms Rating Scale (BSRS-5), measuring respondents’ perceived levels of anxiety (feeling tense or keyed up), depression (feeling depressed or in a low mood), hostility (feeling easily annoyed or irritated), inferiority (feeling inferior to others), and insomnia (having trouble falling asleep) during the past week [41]. Each item is rated on a 5-point Likert scale from 1 (*not at all*) to 5 (*extremely*), with a higher score indicating a higher level of psychological distress. Cronbach’s alphas of this scale were 0.88 at T1 (0.87 for boys and 0.88 for girls) and 0.89 at T2 (0.90 for boys and 0.88 for girls).

Family support. Family support was assessed using four items drawn from a family cohesion scale. Although originally embedded within a broader measure of family cohesion, these items were selected because they capture adolescents’ perceptions of family support across three dimensions: emotional, informational, and instrumental support [42]. The focus on perceived family support was guided by evidence suggesting that perceived support is more consistently associated with mental health outcomes than other dimensions of social support [43]. Family support items include: “When I’m frustrated, I can always seek consolation from my family” (emotional support); “I can rely on my family when I need help or advice” (instrumental and informational support); “Family members discuss things with each other when making decisions” (informational support); and “I feel valued by my family” (emotional support). Each item is rated using a 4-point Likert scale from 1 (*strongly disagree*) to 4 (*strongly agree*), with a higher score representing a higher level of perceived family support. Cronbach’s alphas of this scale were 0.87 at T1 (0.86 for boys and 0.88 for girls) and 0.87 at T2 (0.87 for boys and 0.87 for girls).

Self-efficacy. Self-efficacy was evaluated with the General Self-Efficacy Scale [44]. It consists of 10 items measuring an individual’s perception of his/her ability to manage difficult or unexpected situations. Sample items include “I can always manage to solve difficult problems if I try hard enough” and “Thanks to my resourcefulness, I know how to handle unforeseen situations.” Each item is rated using a 4-point Likert scale from 1 (*Not at all true*) to 4 (*Exactly true*). Cronbach’s alphas for these 10 items were 0.92 at T1 (0.92 for boys and 0.91 for girls) and 0.92 at T2 (0.92 for boys and 0.91 for girls).

### Analysis plan

First, IBM SPSS Statistics for Windows, Version 28.0 was used to calculate the descriptive statistics and bi-variate correlations between variables in this study [45]. Because the present study relied on secondary data and only two waves contained complete information on all focal variables, a half-longitudinal mediation model was adopted as the most appropriate analytic approach for examining the temporal associations among family support, self-efficacy, and mental health outcomes under these data constraints [46–48]. This design evaluates mediation by simultaneously testing the cross-lagged path from the T1 predictor to the T2 mediator together with the path from the T1 mediator to the T2 outcome, while controlling for autoregressive stability and within-wave correlations. This approach incorporates temporal ordering and cross-time effects, and is methodologically superior to cross-sectional mediation, which relies entirely on same-wave associations [46–48].

Structural equation modeling (SEM) was then conducted in AMOS 26.0 to test the hypothesized model [49]. Family support at T1 was specified as the predictor, self-efficacy at both T1 and T2 was included to capture the mediating process, and life satisfaction and psychological distress at T2 were specified as the outcome variables, with autoregressive paths controlling for their corresponding T1 levels. Synchronous correlations among family support, self-efficacy, life satisfaction, and psychological distress were also estimated within each wave. Indirect effects were evaluated using bias-corrected 95% bootstrap confidence intervals based on 5,000 resamples. Model fit was assessed using the normed fit index (NFI), incremental fit index (IFI), comparative fit index (CFI), and root mean square error of approximation (RMSEA). Following conventional criteria, values greater than 0.90 for NFI, IFI, and CFI, and values smaller than 0.08 for RMSEA were considered indicative of satisfactory model fit [50, 51].

To examine whether the structural relationships differed by sex, a multi-group comparison analysis was conducted within the SEM framework. A fully constrained model, in which all structural paths were set equal across male and female groups, was estimated first. Subsequently, each structural path was released one at a time and compared with the fully constrained model. A significant change in chi-square was interpreted as evidence of a sex difference in the corresponding path.

## Results

### Handling of missing data

Prior to conducting the primary analyses, the missing value analysis was conducted. Specifically, missingness was minimal across most study variables, including sex at T1 (0%), family support at T2 (0.73%), self-efficacy (T1 = 0.22%, T2 = 0.90%), life satisfaction (T1 = 0.06%, T2 = 0.62%), and psychological distress (T1 = 0.34%, T2 = 0.73%). The highest missing rate was observed for family support at T1 (5.66%).

Little’s MCAR test was performed to evaluate the missing data [52]. The result indicated that the data were missing completely at random (χ^2^ = 81.016, df = 89, *p* = 0.715). Missing values were addressed using regression imputation via AMOS software before proceeding with the SEM estimations [49, 53].

### Descriptive statistics and bivariate analyses

Among the 1,792 adolescents, more than half (56.9%, *n* = 1020) were female. In Wave 2 (T1), 1,643 participants (91.7%) were students, and 149 participants (8.3%) were employed or stayed at home. In Wave 3 (T2), 1,414 participants (78.9%) were students, and 378 participants (21.1%) were employed or stayed at home. The means and standard deviations of the study variables and bivariate correlations between variables are shown in Table 1. The results show that a high level of family support at T1 was correlated with a high level of self-efficacy (*r* = 0.18, *p* < 0.01) and life satisfaction (*r* = 0.31, *p* < 0.01) and a low level of psychological distress (*r* = −0.21, *p* < 0.01) at T1. Across time, family support at T1 was positively correlated with self-efficacy and life satisfaction (r range = 0.17–0.24, *p* < 0.01) and negatively correlated with psychological distress (*r* = −0.15, *p* < 0.01) at T2. Self-efficacy at T1 was positively correlated with life satisfaction (*r* = 0.17, *p* < 0.01) and negatively correlated with psychological distress (*r* = −0.12, *p* < 0.01) at T2. These results suggest that greater family support at T1 correlated with higher levels of self-efficacy and life satisfaction but lower levels of psychological distress at T2. Higher self-efficacy at T1 correlated with greater life satisfaction and lower levels of psychological distress at T2.

**Table 1:** Descriptive statistics and correlations among all study variables

Variables	M (SD)	1	2	3	4	5	6	7	8
1. T1 Family support	11.88 (2.46)	‒							
2. T2 Family support	11.98 (2.37)	0.53**	‒						
3. T1 Self-efficacy	24.14 (5.64)	0.18**	0.10**	‒					
4. T2 Self-efficacy	24.17 (5.42)	0.17**	0.21**	0.48**	‒				
5. T1 Life satisfaction	20.09 (6.10)	0.31**	0.22**	0.20**	0.12**	‒			
6. T2 Life satisfaction	19.11 (6.00)	0.24**	0.34**	0.17**	0.28**	0.44**	‒		
7. T1 Psychological distress	10.00 (4.42)	−0.21**	−0.15**	−0.16**	−0.09**	−0.30**	−0.22**	‒	
8. T2 Psychological distress	9.80 (4.42)	−0.15**	−0.23**	−0.12**	−0.15**	−0.25**	−0.34**	0.50**	‒

Standard deviations are in parentheses. **p < 0.001, *p < 0.01. T1 = Time 1; T2 = Time 2

### Half-longitudinal mediation model analysis

**Fig. 1:**
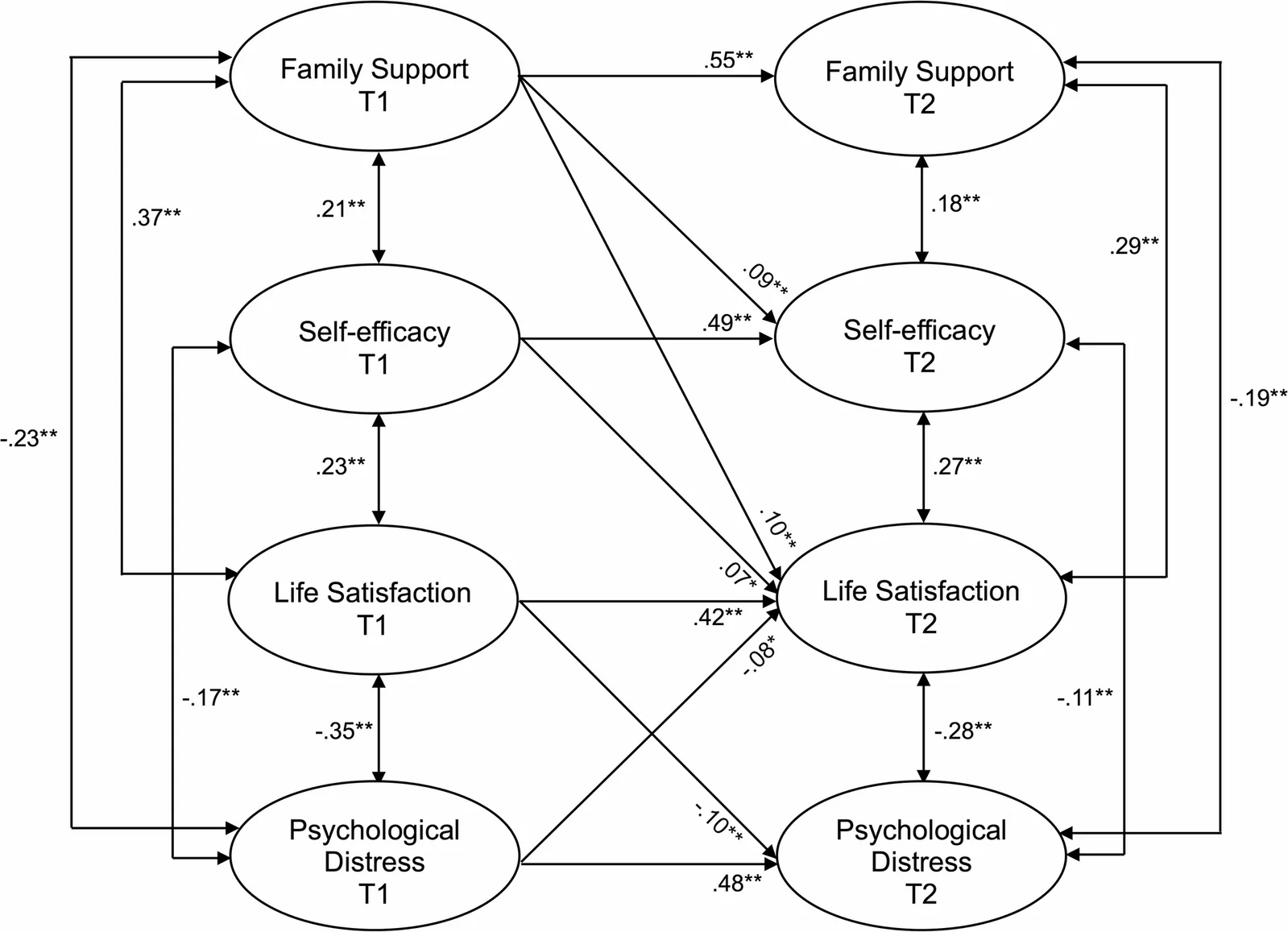
The half-longitudinal mediation model of family support, self-efficancy, life satisfaction and psychological distress. Note: **p < 001. *p < 01

Figure 1 demonstrates the results of the half-longitudinal mediation model, which revealed that this model fit the data well: χ^2^(652) = 2214.5, *p* < 0.001, CFI = 0.96, IFI = 0.96, NFI = 0.95, and RMSEA = 0.037. Within-time correlations between family support, self-efficacy, life satisfaction, and psychological distress were statistically significant at T1 (β = −0.35–0.37, *p* < 0.001). Similarly, at T2, significant within-time correlations were also found between family support, self-efficacy, life satisfaction, and psychological distress (β = −0.28 to 0.29, *p* < 0.001). All autoregressive effects were significant for family support (β = 0.55, *p* < 0.001), self-efficacy (β = 0.49, *p* < 0.001), life satisfaction (β = 0.42, *p* < 0.001), and psychological distress (β = 0.48, *p* < 0.001) across time.

### Direct effects of family support on life satisfaction and psychological distress

Regarding the cross-time association, Fig. 1 indicates that family support at T1 positively predicted life satisfaction at T2 (β = 0.10, *p* < 0.001) but failed to predict psychological distress at T2 in a significant way (β = −0.02, *p* > 0.01). In addition, family support at T1 significantly predicted self-efficacy at T2 (β = 0.09, *p* < 0.001), and self-efficacy at T1 significantly predicted life satisfaction at T2 (β = 0.07, *p* < 0.01) but not psychological distress at T2 (β = −0.03, *p* > 0.01). These findings indicate that family support contributes to positive aspects of mental health (i.e., life satisfaction) but shows no significant effect on negative aspects (i.e., psychological distress).

### Mediating role of self-efficacy

Based on previous studies on the half-longitudinal mediation model [46–48], the indirect effect of family support on life satisfaction through self-efficacy was represented by the product of these two paths. The significant mediation effect was supported by the bias-corrected 95% confidence intervals (CIs) based on 5,000 bootstrap resamples (β = 0.014, CI [0.004, 0.032], *p* < 0.01). In the same way, the indirect effect of family support on psychological distress through self-efficacy was represented by the product of these two paths, and the results were not significant [based on 5,000 bootstrap resamples (β = −0.003, CI [−0.013, 0.003], *p* > 0.01].

In addition, life satisfaction at T1 significantly predicted psychological distress at T2 (β = −0.10, *p* < 0.001), and psychological distress at T1 also predicted life satisfaction at T2 (β = −0.08, *p* < 0.01), suggesting the reciprocal relationship between life satisfaction and psychological distress. Synchronous correlations between life satisfaction and psychological distress were significant at T1 (β = −0.35, *p* < 0.001) and T2 (β = −0.28, *p* < 0.001).

### Sex differences analysis

**Fig. 2:**
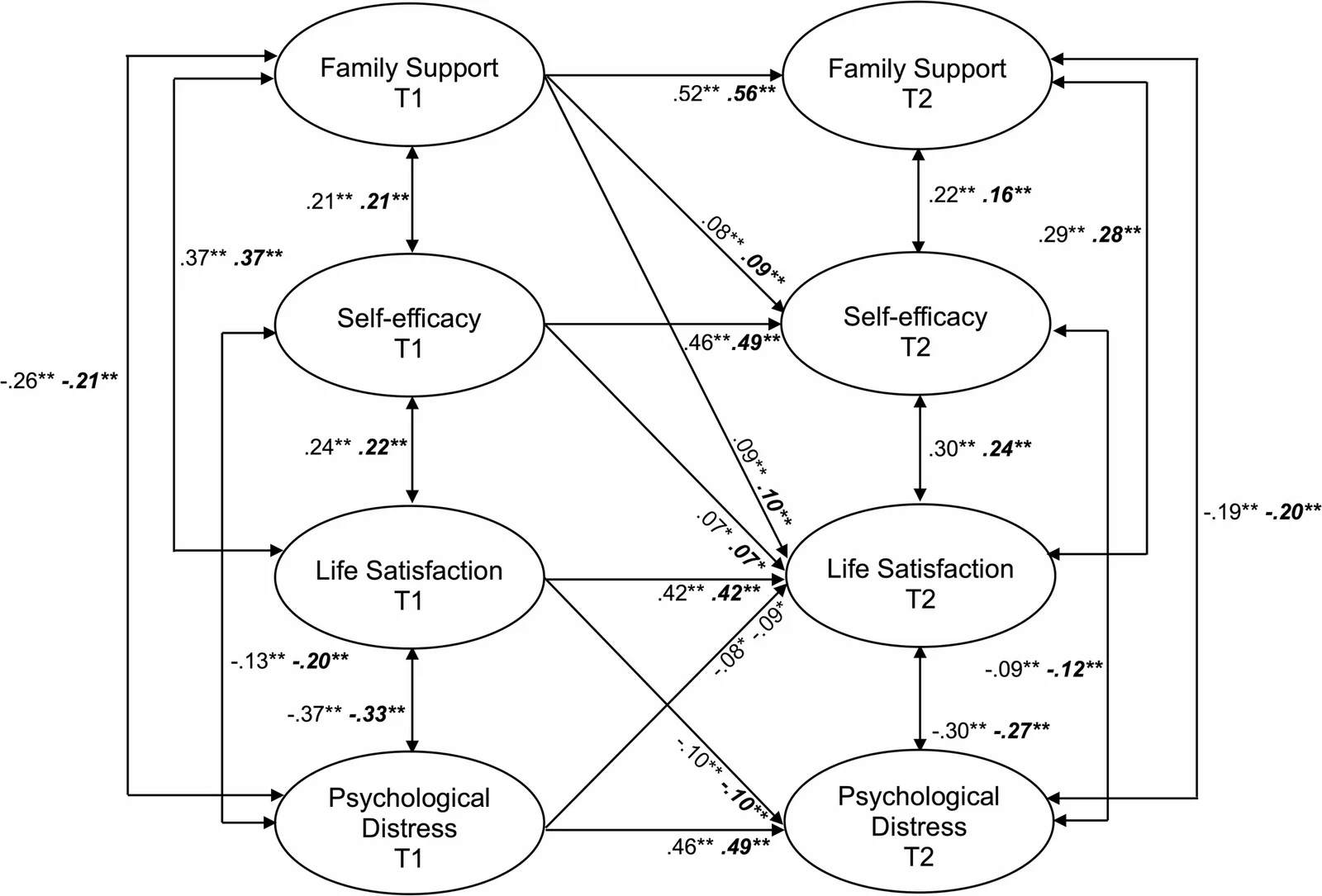
The half-longitudinal mediation model of family support, self-efficancy, life satisfaction and psychological distress between sex groups. The coefficients in regular print and those in bold italics, represent, respectively, the results for boys' and girls' samples. Note: **p < 001. *p < 01

Sex differences were examined using a multi-group comparison analysis within the half-longitudinal mediation model. Figure 2 demonstrates the results of the half-longitudinal mediation model across sexes. Initially, all structural paths between T1 and T2 were constrained to be equal across the boys’ and girls’ groups. The model demonstrated acceptable fit to the data: χ^2^(1252) = 2852.4, *p* < 0.001, CFI = 0.96, IFI = 0.96, NFI = 0.93, and RMSEA = 0.027. Subsequently, to test whether specific relationships varied by sex, each constrained path was released one at a time. The resulting model was compared to the fully constrained model using the chi-square difference test. A significant change in χ^2^ after releasing a particular path was interpreted as evidence of a sex difference in that structural path. The results revealed no significant change in χ^2^ values following release of any of the structural paths within the half-longitudinal mediation model, indicating no statistically significant sex differences in the temporal relationships among family support, self-efficacy, life satisfaction, and psychological distress. Thus, the fully constrained model was retained as the final model for interpretation.

## Discussion

Using two-wave longitudinal data from the TDCYP, this study investigated the mediating role of self-efficacy in the association of family support with mental health as well as sex differences in the tested model. Although the data were collected between 2011 and 2013, the findings remain applicable to the current Taiwanese context. While Taiwan’s social welfare system has evolved from an individual-based subsidy model to a more comprehensive, family-centered approach over the past decade, the cultural emphasis on family values has remained largely unchanged [54]. Within this cultural context, families remain central sources of social support for adolescents, and stable norms centered on family unity and collective expectations continue to shape their experiences. As such, the results of this study retain their relevance and can be meaningfully generalized to the present day.

The present findings differ in part from those reported in previous studies [10, 11, 18, 23, 55, 56], and suggest that the effects of social support on positive and negative mental health may vary across contexts. To be more specific, the current study found that family support was associated with later positive mental health indicator but not with later negative mental health indicator. Given that most previous studies examining the relationship between social support and negative mental health outcomes have been conducted in adolescents from the general population [11, 16, 18, 56], the absence of a significant association between family support and later psychological distress in the present study may reflect the unique challenges faced by socioeconomically disadvantaged adolescents. Adolescents from low-income families are often exposed to multiple stressors, including financial hardship, academic pressures, family conflicts, and other environmental challenges [57–59]. Over time, these stressors may accumulate or become more complex, resulting in a greater risk of psychological distress [60]. Hence, even when adolescents perceive high levels of family support, such support may not be sufficient to offset the adverse effects of cumulative stress exposure on mental health.

Another possible explanation for the absence of a significant association between family support and later psychological distress lies in the dual nature of family relationships in socioeconomically disadvantaged contexts. In Taiwanese society, where family obligations and interdependence are strongly emphasized, family support is often intertwined with expectations of reciprocity and responsibility toward family well-being [24–26]. As a result, adolescents may feel a heightened sense of responsibility for their families. Research suggests that strong family obligation may become a vulnerability factor when adolescents experience economic hardship alongside other negative life events or family stressors [61]. Under such circumstances, adolescents may experiencing additional psychological burden. Consequently, family support may be less effective in reducing later psychological distress.

In contrast, the findings indicate that family support contributes directly to later positive mental health. This result is consistent with previous study findings [10, 14]. Family support may promote psychological well-being by providing adolescents with emotional support, guidance, and a sense of connectedness [11, 12]. In the Taiwanese cultural context, where familism and interpersonal harmony are highly valued, support from family members may further strengthen adolescents’ sense of belonging and relational fulfillment, thereby enhancing life satisfaction [62].

Findings from the half-longitudinal analysis were consistent with an indirect association between family support and life satisfaction through self-efficacy, suggesting that self-efficacy may represent a pathway through which family support contributes to adolescents’ life satisfaction. Family can foster adolescents’ confidence in their ability to cope with challenges and pursue personal goals [18, 19]. In turn, greater self-efficacy may contribute to higher life satisfaction and better mental health by enhancing adolescents’ effective coping abilities in the face of adversity, while fostering the intrinsic motivation and confidence needed to actively pursue and achieve personally valued goals [63].

In the present study, the findings did not support a half-longitudinal indirect association between family support and psychological distress through self-efficacy, suggesting that self-efficacy may not play a prominent role in linking family support to later psychological distress. This finding contrasts with evidence from adolescents from the general population [11, 16, 56]. The discrepancy may reflect the more complex and persistent challenges faced by adolescents from socioeconomically disadvantaged families. In addition to financial hardship, economic disadvantage may contribute to a range of family and interpersonal stressors, including elevated parental stress, greater family conflict, and difficulties in peer relationships, all of which have been linked to psychological distress [2, 3, 5]. Within the Taiwanese cultural context, where interpersonal harmony, family connectedness, and relational obligations are highly valued, psychological distress may be more closely tied to interpersonal and family-related difficulties than to adolescents’ achievement of personal goal [18, 62, 64]. Although self-efficacy may enhance adolescents’ confidence in managing challenges and pursuing personal goals, it may be less effective in addressing stressors that arise from strained family relationships or broader social circumstances. Consequently, the role of self-efficacy in reducing psychological distress may be more limited among socioeconomically disadvantaged adolescents in Taiwan.

Taken together, although previous cross-sectional studies [10, 11, 18, 23, 55] have consistently reported associations between family support and both positive and negative mental health outcomes, the present findings suggest that family support may play a stronger role in promoting positive mental health than in alleviating negative mental health over time. Moreover, self-efficacy may partially explain how family support is associated with later life satisfaction. This pattern indicates that positive and negative mental health may be shaped by different mechanisms.

Finally, the present study found no evidence of sex differences in the pathways linking family support, self-efficacy, and mental health. Although previous studies have suggested that self-efficacy may play a more prominent role in boys’ adjustment due to their greater emphasis on competence and goal attainment [38], the current findings indicate that the influence of family support and self-efficacy on mental health operates similarly for both males and females. This finding suggests that family support and self-efficacy may serve as important mental health resources regardless of sex. Nevertheless, the absence of statistically significant differences does not necessarily imply that intervention approaches should be identical for boys and girls.

### Practice implications

The findings suggest that, for adolescents and youth in low-income families in Taiwan, policies and interventions should include not only material assistance but also psychosocial strategies that promote positive mental health. While provision of material resources could address survival needs and relieve stress due to financial strain, psychosocial interventions might have longer-term effects on well-being. As family support in Taiwanese culture is closely tied to expectations of familial obligation, a pattern shared across many Chinese family systems [29], these policy implications may extend to other Chinese societies.

In alignment with the current shift in Taiwan’s social services toward holistic, family-based psychosocial support, the present findings have important implications for practice. Given the importance of family support and self-efficacy for adolescents’ positive mental health, and considering the Taiwanese cultural context in which family support is often accompanied by high expectations and protective parenting, interventions should go beyond simply increasing support and also address underlying family dynamics. In particular, autonomy-supportive and strengths-based approaches should be applied to strengthen adolescents’ self-efficacy [65, 66]. Policymakers and service providers may integrate these interventions into existing financial assistance programs and social services serving low-income families by providing parents with family psychoeducation and parent–adolescent communication programs. Schools and community family service centers may also serve as important settings for delivering additional family-based interventions that incorporate autonomy-supportive and strengths-based approaches.

At the same time, youth-focused interventions are equally important. Because perceived support is shaped not only by the support provided but also by how it is interpreted by the recipient [67], helping adolescents recognize, communicate, and seek support for their specific needs is particularly important. Accordingly, community youth services may incorporate interventions that equip adolescents from low-income families with the skills to navigate familial expectations, communicate their psychological needs effectively, and actively seek appropriate support. In addition, given the positive association between self-efficacy and later positive mental health, schools could provide brief strengths-based interventions that help adolescents from low-income families identify personal strengths, set achievable goals, and develop confidence in their ability to overcome challenges and achieve personal goals. Such interventions may further strengthen self-efficacy and promote positive mental health.

### Limitations

Despite its important implications, this study has several limitations. First, although longitudinal data can inform temporal ordering, our reliance on secondary data constrained the design. As only two waves of data were available, we used a half-longitudinal mediation model for the analysis. Studies with three or more waves are needed to identify longitudinal relations and indirect effects more precisely. Second, the interval between waves (i.e., 2 years) was relatively long. Because BSRS-5 assesses distress during the past week, the present findings should be interpreted as reflecting prospective associations in psychological distress across a 2-year developmental interval rather than continuity of a specific acute symptom episode. Similarly, self-efficacy may fluctuate during adolescence, and because only two waves were available, the present study could not fully separate short-term fluctuations from longer-term stability. Accordingly, future work should employ tighter intervals and additional waves to test the model’s applicability across time scales and to more precisely capture developmental changes in self-efficacy. Third, our sample comprised adolescents in Taiwan, so generalizability to Western adolescents from low-income families remains to be established. Fourth, due to the use of secondary data analysis, the measurement of family support was derived from a broader family cohesion scale available in the dataset. Although the selected items were theory-driven, reflecting key dimensions of perceived support, and showed good internal consistency across both waves, they were not drawn from a standardized family support measure. Future studies could use validated and comprehensive measures to improve construct precision. In addition, future research could further investigate how different dimensions of family support influence positive and negative mental health over time. Finally, as the current study relied on self-report measures, the possibility of common method bias, including influences such as social desirability and mood-state effects, cannot be ruled out. Given that this was a secondary data analysis, the dataset lacked relevant variables to statistically control for such bias. However, indirect indicators imply that its impact may be constrained. For example, the distribution of key variables showed no evidence of extreme skewness or ceiling effects, and the data were approximately normally distributed, indicating that responses were not uniformly biased toward socially desirable reporting. In addition, the differential pattern of associations observed across positive and negative mental health outcomes suggests that mood-state effects are unlikely to be the primary driver of the findings. Yet, future studies should incorporate multi-informant or objective measures, or include variables that allow for the control of potential biases, to strengthen the validity of these findings.

## Conclusions

Drawing on longitudinal data, this study examined how family support influences both positive and negative dimensions of mental health among Taiwanese adolescents from low-income families. The results indicated that greater family support predicts improved later life satisfaction, but not later psychological distress. Moreover, the findings were consistent with a half-longitudinal indirect association between family support and life satisfaction through self-efficacy. There was no sex difference in the mediating role of self-efficacy in the relationship between family support and mental health. These findings suggest that the effects of family support are not uniform across positive and negative dimensions of mental health. Policies and interventions aimed at promoting mental health in this population should prioritize strengthening self-efficacy.

## Data Availability

The present analyses were based on the Taiwan Database of Children and Youth in Poverty collected in Taiwan in 2011 and 2013. Data and codebook are available from https://srda.sinica.edu.tw/plan/?idx=SRDA.AS021
